# Supplementary material for: Splice-Junction-Based Mapping of Alternative Isoforms in the Human Proteome
Source: Cell Rep. Author manuscript; Available in PMC 2020 Jan 15. (PMC6961840; doi:10.1016/j.celrep.2019.11.026)
Supplement: 2 [file NIHMS1546469-supplement-2.zip › DF1/PXD006675/CardiacFibroblast/CardiacFibroblast_11_SYNRG_FSTASASHSVPEAGPSLEEK.pdf]

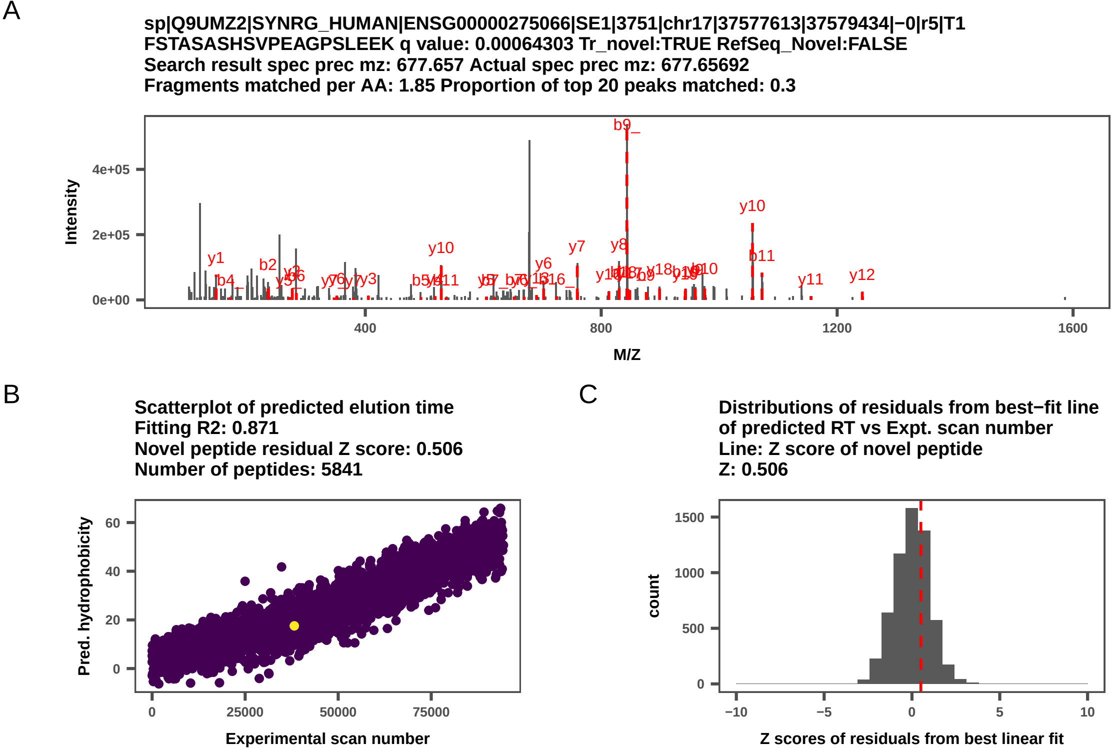

A
sp|Q9UMZ2|SYNRG_HUMAN|ENSG00000275066|SE1|3751|chr17|37577613|37579434|−0|r5|T1
FSTASASHSVPEAGPSLEEK q value: 0.00064303 Tr_novel:TRUE RefSeq_Novel:FALSE
Search result spec prec mz: 677.657 Actual spec prec mz: 677.65692
Fragments matched per AA: 1.85 Proportion of top 20 peaks matched: 0.3
Intensity
4e+05
2e+05
0e+00
M/Z
400
800
1200
1600
y1
b2
y10
y7
y8
y6
y10
b11
y11
y12
B
Scatterplot of predicted elution time
Fitting R2: 0.871
Novel peptide residual Z score: 0.506
Number of peptides: 5841
Pred. hydrophobicity
60
40
20
0
0
25000
50000
75000
Experimental scan number
C
Distributions of residuals from best−fit line
of predicted RT vs Expt. scan number
Line: Z score of novel peptide
Z: 0.506
count
1500
1000
500
0
−10
−5
0
5
10
Z scores of residuals from best linear fit
